# The Mitochondrial Proteome of Tumor Cells: A SnapShot on Methodological Approaches and New Biomarkers

**DOI:** 10.3390/biology9120479

**Published:** 2020-12-18

**Authors:** Loredana Moro

**Affiliations:** Institute of Biomembranes, Bioenergetic and Molecular Biotechnologies, National Research Council, Via Amendola 122/O, 70125 Bari, Italy; l.moro@ibiom.cnr.it

**Keywords:** mitochondria, cancer, mitochondrial proteome, mass spectrometry, immunofluorescence, interactome

## Abstract

**Simple Summary:**

Mitochondria are central hubs of cellular signaling, energy metabolism, and redox balance. The plasticity of these cellular organelles is an essential requisite for the cells to cope with different stimuli and stress conditions. Cancer cells are characterized by changes in energy metabolism, mitochondrial signaling, and dynamics. These changes are driven by alterations in the mitochondrial proteome. For this reason, in the last years a focus of basic and cancer research has been the implementation and optimization of technologies to investigate changes in the mitochondrial proteome during cancer initiation and progression. This review presents an overview of the most used technologies to investigate the mitochondrial proteome and recent evidence on changes in the expression levels and delocalization of certain proteins in and out the mitochondria for shaping the functional properties of tumor cells.

**Abstract:**

Mitochondria are highly dynamic and regulated organelles implicated in a variety of important functions in the cell, including energy production, fatty acid metabolism, iron homeostasis, programmed cell death, and cell signaling. Changes in mitochondrial metabolism, signaling and dynamics are hallmarks of cancer. Understanding whether these modifications are associated with alterations of the mitochondrial proteome is particularly relevant from a translational point of view because it may contribute to better understanding the molecular bases of cancer development and progression and may provide new potential prognostic and diagnostic biomarkers as well as novel molecular targets for anti-cancer treatment. Making an inventory of the mitochondrial proteins has been particularly challenging given that there is no unique consensus targeting sequence that directs protein import into mitochondria, some proteins are present at very low levels, while other proteins are expressed only in some cell types, in a particular developmental stage or under specific stress conditions. This review aims at providing the state-of-the-art on methodologies used to characterize the mitochondrial proteome in tumors and highlighting the biological relevance of changes in expression and delocalization of proteins in and out the mitochondria in cancer biology.

## 1. Introduction

Mitochondria are highly dynamic organelles historically considered as the energetic powerhouse of the cell. Besides fueling vital cellular processes with ATP, mitochondria modulate cell proliferation, fatty acid metabolism, iron homeostasis, autophagy, programmed cell death, reactive oxygen species (ROS) generation, and the storage/flux of the second messenger calcium [1,2,3,4,5]. In addition, mitochondria represent an anti-viral signaling hub that activates the immune response through the mitochondrial antiviral signaling (reviewed in [6,7]). Owing to the role of mitochondria in a wide array of cell functions, it is not surprising that dysregulated mitochondrial processes have been linked at various degree to development and malignant progression of human cancers [8,9]. 

From an evolutionary point of view, mitochondria originate from the endosymbiotic fusion of an Alphaproteobacteria with a primordial ancestral cell [10,11]. Over time, much of the genetic information of bacterial origin was lost or relocated to the host nucleus through lateral gene transfer [12,13], leading to the formation of mitochondria as cell organelles. Traits of the endosymbiotic origin of mitochondria are the presence of mitochondrial ribosomes, a mitochondrial genome, the phospholipid cardiolipin and β-barrel proteins in the outer membrane, which are a bacterial peculiarity. Notably, mitochondria use a genetic code that differs from the universal code [14]. Structurally, mitochondria are composed of two membranes: an outer mitochondrial membrane (OMM) and an inner mitochondrial membrane (IMM) that demarcate the intermembrane space (IMS). The IMM forms large invaginations, termed cristae, which are the sites where protein complexes of the respiratory chain are localized. The IMM delimits an aqueous internal compartment, called matrix, which contains several components, including the enzymes of the Krebs’ cycle, the mitochondrial ribosomes, and the mitochondrial genome (mtDNA). Sequencing of the 16,569 bp human mtDNA in 1981 represented an important milestone in mitochondrial biology [15]. A key information derived from mtDNA sequencing is that the mitochondrial genome contains limited coding capacity and thus can only account for a small proportion of the mitochondrial proteome. Indeed, the mitochondrial proteome comprises about 1000 proteins in *Saccharomyces cerevisiae* and at least 1500 proteins in humans [16]. The 13 proteins encoded by the human mtDNA are essential components of the mitochondrial respiratory chain and represent about 10% of the mitochondrial respiratory proteins [16]. All the remaining mitochondrial proteins, including enzymes of the Krebs’ cycle, apoptotic proteins, transport proteins, and the other proteins of the respiratory chain complexes, are encoded by the nuclear genome, thus they have to be imported into mitochondria.

There are several mechanisms involved in the import of proteins into mitochondria (reviewed in [17]). A first transport mechanism directs proteins into the mitochondrial matrix through an N-terminal cleavable amino acid pre-sequence, which interacts with membrane-bound proteins belonging to the multi-protein complex translocon of the outer mitochondrial membrane (TOM) [18]. The interaction of TOM proteins with different elements of this pre-sequence allows a directional cross of the outer membrane from the N- to the C-terminus of the protein [19]. Once in the IMS, proteins of the translocon of the inner membrane complex (TIM) interact with the pre-sequence allowing protein transfer in the matrix. This transfer is driven by the electrochemical gradient (ΔΨ) across the IMM [20,21]. In the mitochondrial matrix, the N-terminal pre-sequence is cleaved, and the protein is folded by matrix chaperones of the Hsp60 family [22,23]. A second mechanism of protein import involves anchoring of proteins to the OMM through N-terminal α-helics in an N-to-C-terminal or a C-to-N-terminal orientation [24]. The import of proteins in the IMM can occur via interaction with the TIM23 or TIM22 complex or proteins are first transported in the matrix and then integrated in the IMM (reviewed in [25]). Finally, proteins in the IMS have a short sequence rich in cysteine residues that are oxidized to disulfide bonds upon import through interaction with Mia40 [26,27].

Mitochondria undergo constant changes in number and morphology through fusion/fission dynamics during cell division, to adapt to the cell energetic needs and to respond to extracellular stimuli/stresses. As such, mitochondrial dynamics regulate physiological and pathological processes, including apoptosis, metabolic programming/reprogramming, and the overall mitochondrial functionality (reviewed in [28]). During fusion, the OMM and IMM of two mitochondria fuse with each other through a process regulated by dynamin related GTPases Mitofusin (MFN) 1 and 2, and by optic atrophy 1 (OPA1). MFN1 and MFN2 coordinate OMM fusion, whereas OPA1 coordinates IMM fusion [29,30]. Mitochondrial fission results in fragmentation of pre-existing mitochondria and requires full cooperation and interaction with the endoplasmic reticulum (ER). ER in contact with mitochondria marks the position where mitochondria must split off. Dynamin-related protein 1 (DRP1), a cytosolic protein, is recruited to the mitochondrial surface by binding to OMM transmembrane receptors, including MFFs, FIS1, MID49/51. On the OMM, DRP1 forms oligomeric complexes and mediates the scission of mitochondria. Notably, its activity is regulated by post-translational modifications, including ubiquitination, sumoylation, and phosphorylation. In general, mitochondrial fusion promotes the oxidative metabolism whereas the presence of fragmented mitochondria is associated with anaerobic glycolysis (for a detailed description of mitochondrial fusion and fission see [31]). Imbalanced mitochondrial dynamics has been implicated in tumor pathogenesis (reviewed in [32]). For example, hypoxia-induced increase of mitochondrial fission in ovarian cancer cells induces cisplatin resistance [33]. Similarly, the presence of fragmented mitochondria in breast cancer cells has been linked to their resistance to tamoxifen [34].

Mitochondrial dynamics is also strictly intertwined with mitophagy, the specific autophagic disruption of mitochondria [35,36]. The mitochondrial genome is highly exposed to ROS, being mitochondria the main sites of ROS production through the respiratory chain. This results in the accumulation of mtDNA mutations that can impair the mitochondrial functionality and, thus, the cell survival. The activation of mitophagy occurs when mitochondria are damaged beyond the ability of other quality control pathways or when the cell has to remove superfluous mitochondria to meet metabolic or developmental needs in response to a plethora of stimuli and triggers [35,37]. Two pathways regulate mitophagy: the phosphatase and tensin homologue (PTEN)-induced putative kinase 1 (PINK1)-PARKIN pathway and the PARKIN-independent pathway. Mitochondrial fission plays a crucial role in mitophagy as it is preceded by division of mitochondria. Fragmented mitochondria are then incapsulated by the autophagosomes [35]. Accordingly, the overexpression of OMM transmembrane receptors for DRP1 triggers mitophagy [38]. Similarly, PINK1, by indirectly interacting with DRP1, promotes its activation, fostering mitochondrial fission and impairing mitochondrial fusion [39]. 

Besides interacting with ER during fission, mitochondria actively cooperate with peroxisomes and the nucleus via signal transduction and vesicle transport (for excellent recent reviews see [4,40]). Briefly, cooperation between peroxisomes and mitochondria is important to maintain the equilibrium of ROS levels through scavenging enzymes, for lipid synthesis, and to counteract viral infections. Mitochondria and peroxisomes share many proteins involved both in their biogenesis, like PGC1α, and fission, like FIS1 and MFF (reviewed in [40]). Mitochondrial-nuclear cooperation and coordination are mediated by bidirectional transmission pathways, namely the anterograde (nucleus to mitochondria) and the retrograde (mitochondria to nucleus) signaling pathways (reviewed in [4,41]). The retrograde signaling includes the exchange of mitochondrial- and nuclear-encoded factors/co-factors, and metabolic intermediates that can elicit epigenetic modifications. Among several mitochondrial-derived signaling molecules, including NAD+, ROS and calcium, it is noteworthy to mention the recent discovery of eight small bioactive peptides encoded by short open reading frames (ORFs) within the mtDNA, collectively referred to as mitochondrial-derived peptides (MDPs) [42]. MDPs include humanin, six small humanin-like peptides, and mitochondrial ORF of the 12S ribosomal RNA type-c (MOTS-c). Notably, in response to metabolic stress, MOTS-c is delocalized to the nucleus thereby interacting with transcriptional factors and regulating adaptive nuclear gene expression [43]. This is the first mitochondrial-encoded factor shown to directly regulate the nuclear genome. Another layer of increased complexity in the retrograde signaling is represented by the recent discovery that the transcriptional regulator GPS2 shuttles from mitochondria to the nucleus of mammalian cells following mitochondrial depolarization [44]. The delocalization of GPS2 from the OMM to the nucleus elicits transcriptional activation of nuclear-encoded mitochondrial genes and other stress-response genes. It also sustains basal mitochondrial biogenesis during adipogenesis [44]. Finally, the ER-mitochondria interaction goes beyond the fission process described above. Stable bridges between ER and mitochondria coordinate calcium signaling, ER stress response, apoptosis, and transfer of ER-synthetized phospholipids to the mitochondrial membranes. The tight contacts between the ER and mitochondria constitute the mitochondria-associated membranes (MAMs) and are a hub of signaling proteins, tumor suppressors and oncogenes regulating cell survival and growth (for excellent reviews on this topic see [40,45]). Among others, the oncogene BCL-2 is highly enriched at MAMs, both on the ER and the mitochondrial side, where it acts as a cytoprotective, antiapoptotic protein. On the ER side, BCL-2 prevents the mobilization of calcium towards mitochondria by interacting with IP3R to prevent channel opening [46]. On the OMM, BCL-2 binds to the proapoptotic BAX/BAK proteins preventing their oligomerization and formation of the “apoptotic pore” [47,48]. On the other hand, the expression of tumor suppressor proteins like BRCA2, PTEN, and PML increases calcium transfer from ER to mitochondria thereby promoting apoptosis [49,50,51]. Notably, the loss of PML confers resistance to chemotherapy due to calcium transfer from ER to mitochondria that promotes autophagy and confers a metabolic a survival advantage to tumor cells sustaining cancer development [52]. The interaction between ER and mitochondria also occurs during the ER stress response, a common stress due to accumulation of unfolded proteins that triggers a conserved pathway termed the unfolded protein response (UPR). During UPR, mitochondria provide energy for protein folding in the ER and activate apoptosis if the ER stress persists. Unmitigated ER stress can profoundly affect mitochondrial function by causing protein overload in mitochondria [53,54]. A recently identified 54-amino acid mitochondrial microprotein, PIGBOS, has been implicated in the regulation of UPR [55]. PIGBOS localizes to the OMM at the ER-mitochondria contact sites and its loss increases UPR and apoptosis [55]. To date, PIGBOS is the only mitochondria-specific protein shown to modulate UPR in the ER.

As mentioned above, the exact composition of the mitochondrial proteome may vary depending on specific stimuli, triggers or stresses that, for example, can activate fission versus fusion, or promote mitophagy. Tumor cells may exhibit altered expression of proteins involved in mitochondrial metabolism, mitochondrial dynamics, mitophagy, and mitochondrial communication with other organelles [1,32,36,44,45,51,56,57]. One of the most striking examples is the overexpression of the OMM protein BCL-2 that fosters the malignant transformation and progression of certain cells of the hematopoietic system by inhibiting apoptosis. This leads to increased cell survival and promotes lymphomagenesis [58]. In addition, BCL-2 overexpression can render lymphoma cells refractory to chemotherapy owing to its anti-apoptotic function [59]. Other examples directly implicate mitochondrial energy metabolism in tumorigenesis. Pathogenic mutations of nuclear genes encoding for the enzymes of the Krebs’ cycle succinate dehydrogenase, fumarate hydratase, and isocitrate dehydrogenase predispose to development of some tumors, including hereditary paraganglioma, leiomyomas, and papillary renal cell cancer (reviewed in [60]). Loss of these mitochondrial enzymes would promote metabolic reprogramming which fuels tumor cell growth and proliferation [61]. Investigating whether and how alterations of the mitochondrial proteome are involved in tumor pathogenesis has relevance in cancer research because it may improve our understanding of the molecular bases of cancer development and progression, and potentially provide new diagnostic and prognostic biomarkers as well as innovative molecular targets to be explored for anti-cancer treatment. Recent advances in mass spectrometry (MS), genomics, and bioinformatics prediction analysis have made possible the identification of more than 1500 proteins that constitute the human mitochondrial proteome. The aim of this review is to provide an update on strategies used to characterize the mitochondrial proteome and, in the second part, to highlight recent examples of proteins found to localize to mitochondria or delocalize from mitochondria to other organelles in human cancer cells. The significance of these changes in the context of tumor biology and the potential clinical implications is discussed.

## 2. Methodologies to Study the Mitochondrial Proteome

There is currently no single established method that can lead to identification of the complete set of mitochondrial proteins. Instead, a combination of several methodologies is considered the best strategy to discover new mitochondria-localized proteins as well as to confirm localization of specific proteins in these organelles. 

Mitochondria are characterized by a complex structure with sub-compartments deputed to different functions. This highlights the importance of not only defining the mitochondrial localization of a protein, but also of identifying its precise compartmentalization, thus adding further complexity to the study of the mitochondrial proteome. In addition, hydrophobic proteins localized in the membranes may be under-represented when using conventional subcellular fractionation methods. For this reason, mitochondria subfractionation as well as optimized membrane solubilization methods may be required to investigate the mitochondrial proteome. 

### 2.1. Mass Spectrometry (MS)

Advancements in MS techniques have allowed a rapid increase in the list of proteins assigned to the mitochondrial compartment. However, this experimental approach has some limitations and challenges. One of these challenges is to distinguish contaminants of the mitochondrial preparation from real mitochondria-localized proteins. Another limitation is the sensitivity of the technique: low-abundant and highly hydrophobic proteins may be under-represented and often missed in MS analyses [62]. For example, many mitochondrial proteins are membrane-localized, and these proteins are difficult to identify by MS because most purification protocols are aimed at extracting soluble proteins. 

#### 2.1.1. Isolation of Mitochondria

The first step in the study of the mitochondrial proteome by MS is the isolation of pure mitochondria. This can be accomplished by using commercially available kits or a manual procedure. Detailed protocols as well as pros and cons of each method have been widely described in the literature [3,63,64,65,66,67]. Briefly, the protocols to isolate mitochondria involve two main steps: cell permeabilization and plasma membrane disruption to release mitochondria and other cell structures, and differential centrifugation to recover fractions enriched for mitochondria. The principle of differential centrifugation is the separation of different biological structures by their sedimentation coefficient, a factor that takes into account density and shape. Different centrifugal forces applied to biological samples in buffered salt solutions with particular density will cause structures with a similar sedimentation coefficient to “sediment” at the bottom of a centrifugation tube at the same time. The cell structures in suspension may be centrifuged at higher centrifugal force to remove other components and so on, until a pure biological fraction is obtained. Each tissue type and cell line may need optimization of the extraction protocol, particularly when the yield is low as in the case of mitochondria from brain regions. As an example, a novel strategy based on fractionated mitochondrial magnetic separation that employs magnetic anti-TOM22 antibodies, has been developed to isolate functional synaptic and non-synaptic mitochondria from murine cortex and hippocampus [68]. Methods have also been developed to isolate the IMM and the OMM [69,70]. 

#### 2.1.2. Discovery-Based MS Approaches

The first mitochondrial proteomic screening, which was performed in 1998 on human placental mitochondria, detected 46 mitochondrial proteins but many of these were contaminant cytoskeletal proteins [71]. In this first MS analysis, the technical approach consisted of 2D-PAGE followed by peptide mass fingerprinting or N-terminal microsequencing. In 2003, the purification of mitochondria from human neuroblastoma cells using a metrizamide gradient followed by 2D-PAGE coupled with MS (matrix-assisted laser desorption/ionization [MALDI]-MS) led to the identification of 185 proteins, of which 132 were mitochondrial, 23 were assigned to the endoplasmic reticulum, peroxisomes or cytosol, and 30 were unknown gene products [72]. In the same year, Taylor et al. identified 615 distinct proteins in human heart mitochondria purified with a metrizamide gradient centrifugation followed by solubilization with N-dodecyl-β-D-maltoside and analyzed by liquid chromatography-tandem mass spectrometry (LC-MS/MS) [73]. In the following years, technical improvements in mitochondria purification together with technological advances in proteomic analyses have allowed identification of about 2000 mitochondrial proteins. Excellent reviews on this topic have been recently published [3,74]; here I will briefly summarize some novel relevant examples of MS techniques to discover new proteins. 

Chemical labeling using isobaric tags for relative and absolute quantification (iTRAQ) and tandem mass tag (TMT) reagents are two similar techniques mostly used in proteomics to investigate clinical samples, particularly plasma and serum specimens, to search for novel cancer biomarkers [75]. These techniques have been recently used to analyze the mitochondrial proteome of cancer tissues. For example, Li N. et al. used iTRAQ quantitative proteomic to analyze the mitochondrial proteome and phospho-proteome of ovarian cancers and control ovarian tissues [76,77]. In the first screening, they identified approximately 5000 proteins from purified mitochondrial samples: 262 of them were associated with overall survival of ovarian cancer patients and 63 were identified as potential ovarian cancer biomarkers [76]. In a follow-up paper, using an iTRAQ-TiO_2_ enrichment-LC-MS/MS experimental approach the authors identified 67 mitochondrial proteins that were phosphorylated in control and ovarian cancer samples. Among them, 48 proteins were differentially phosphorylated in cancer compared to controls [77]. 

#### 2.1.3. Strategies to Quantify Mitochondrial Proteins by MS 

A widely used labeling method that can be applied to define the proteome in cell cultures but not in clinical samples is the stable isotope labeling by amino acids in cell culture (SILAC). This method relies on the incorporation of non-radioactive, stable isotope-containing amino acids during protein synthesis. The method introduces a mass difference between two proteomes that can be distinguished by MS [78]. 

MS-based targeted proteomic approaches have emerged in the last years to quantify predefined, specific proteins, or their post-translational modifications (PTM). These approaches comprise selected/multiple reaction monitoring (SRM/MRM) and parallel reaction monitoring (PRM) [79]. These approaches are useful to quantify known mitochondrial proteins in cells and tissues under different pathological or developmental conditions/stages but not to discover new mitochondrial proteins. So far, a targeted proteomic approach to characterize the human mitochondrial proteome has been applied only in a few studies. A study used SRM to analyze the mitochondrial proteome in the cardiac tissue of patients with dilated cardiomyopathy [80]. The authors showed alterations in several mitochondrial proteins involved in energy metabolism and found that overexpression of the antioxidant thioredoxin-dependent peroxide reductase PRDX3 was significantly associated with impaired ventricular activity. Wolters et al. successfully used a SRM approach to measure over 50 human mitochondrial proteins covering the Krebs’ cycle, OXPHOS, fatty acid β-oxidation, and ROS detoxification in isolated mitochondrial fractions from cultured fibroblasts and total liver extracts [81]. Lam et al. developed a phospho-MRM approach to quantify endogenous phosphopeptides belonging to 11 mitochondrial proteins, including the OMM protein voltage-dependent anion channel (VDAC) and proteins localized in the IMM, such as ANT and members of Complexes I, III, and V, in mitochondria isolated from mouse heart [82]. More recently, Harper’s group developed a PARKIN target-PRM approach to quantify site-specific ubiquitination and abundance of 15 proteins of the OMM during the early phases of PARKIN-dependent mitophagy [83]. This approach was first tested in the HeLa cancer cell line and then confirmed in human embryogenic stem cell-derived induced neurons, providing a potential innovative tool to study the molecular basis of neurodegenerative processes, including Parkinson’s and Alzheimer’s diseases. Taken together, these studies highlight the potential of targeted proteomics to understand the dynamics of the mitochondrial proteome and its posttranslational modifications in various pathological conditions. The advantage of targeted versus large-scale quantitative proteomics aimed at discovering new mitochondrial proteins lays in the possibility to analyze low-abundant proteins. The disadvantages are that: a) the targeted proteomic approach requires previous knowledge of the peptides and b) a relatively small number of proteins can be analyzed, compared to large-scale proteomic analysis. Among the targeted proteomics approaches, SRM/MRM is actually the most common technique used in clinical proteogenomic applications, owing to its sensitivity and relatively low cost. 

### 2.2. Reverse Phase Protein Array (RPPA)

RPPA is a quite novel, sensitive, and high-throughput proteomic analysis that allows quantitative analysis of the differential expression of known proteins as well as of their post-translational modifications (phosphorylation, acetylation, cleavage). Small amount of protein lysates (picograms to femtograms of protein) derived from a variety of biological samples, including tissues, cultured cells and body fluids, are printed per array slide, and then incubated with the specific primary antibody. This technology was first used, and the term coined by Paweletz et al. [84] to analyze changes in cell signaling in pre-malignant prostate lesions. RPPA is dependent on the availability of antibodies able to detect with high affinity and specificity a protein or its post-translational modified version [85]. This technology has been applied in functional proteomic studies of a large number of cell and tissue extracts obtained from a relatively small number of cells to analyze both nuclear- and mitochondrial-DNA encoded proteins. 

### 2.3. The Ascorbate Peroxidase (APEX) Approach

Traditional MS-based proteomic approaches are applied for cellular compartments that can be purified in high yield. To characterize the proteome of cellular regions difficult to purify, an engineered APEX has been developed [86,87]. APEX is first genetically engineered to target a specific cellular compartment/subcompartment in living cells. Upon the addition of biotin-phenol and hydrogen peroxide (H_2_O_2_), APEX catalyzes the covalent binding of the biotin-phenoxyl radical oxidation product to endogenous proteins. Upon cell lysis, biotinylated proteins are pulled down using streptavidin beads and identified by MS. APEX has been used to map the proteome of the human mitochondrial matrix [87]. This strategy identified 495 specific proteins of the matrix, including 31 novel proteins not previously localized into mitochondria. A modified methodology based on ratiometric tagging of endogenous proteins by APEX in the IMS compared with APEX in the cytosol and subsequent data filtering, has been applied to define the IMS proteome in HEK 293T cells [88]. The modification of the traditional APEX was necessary because cytosolic proteins can shuttle in the IMS through the OMM and be not specific of this cell compartment. The modified APEX approach identified 127 proteins in the IMS with >94% specificity [88]. More recently, Hung V. et al. [89] have been able to characterize 137 OMM proteins facing the cytosol using peroxidase-mediated proximity biotinylation in human fibroblasts [90] followed by MS. This approach has greatly enriched the inventory of proteins localized on the OMM. 

### 2.4. MS-Based Methods to Analyze the Mitochondrial Protein Interactome

Mitochondrial function and dynamics are exquisitely regulated by protein interaction networks. A traditional approach to identify protein interactors is the combination of affinity purification followed by MS (AP-MS). The protein under study (bait) is tagged with an affinity tag such as FLAG-tag, HA-tag, GFP-tag, and expressed in cells. The proteins interacting with the bait are then pulled down by affinity techniques and analyzed by MS [91]. This basic approach has been applied to characterize a number of mitochondrial complexes, including the ATPase protein components [91] and the BCL-2 interactome in lung cancer cells [92]. However, this method has some limitations because it does not allow the study of system-wide organization of proteins in mitochondria, and protein tagging can disrupt some interactions [93]. 

Cross-linking coupled with MS (XL-MS) has provided a detailed picture of system-wide organization of proteins in intact mitochondria [94,95]. This technology is based on the capture of native protein contacts using a cross-linker, usually an organic molecule consisting of a spacer arm and two functional groups that react with residue side chains. Isolated mitochondria are cross-linked, lysed, digested with trypsin, and subjected to MS-based peptide sequencing. This approach has provided direct physical evidence of the assembly of the Complex I-III respirasome [95] and has supported the existence of the Complex I-V supercomplex assembly [94]. In the future, large-scale chemical cross-linking analyses may enhance our knowledge on multi-protein complexes and conformational changes in dynamic native conditions, including changes of supercomplexes composition/conformation in response to chemotherapies or radiation.

### 2.5. Immunofluorescence 

Microscopy is a complementary approach to establish and/or confirm mitochondrial localization of a protein. It relies on the availability of antibodies to detect endogenous proteins. Alternatively, proteins can be visualized in intact cells by tagging them with a fluorescent probe or with a tag that can be detected with an already validated antibody (a-FLAG, a-HA, a-GFP, etc.). In the last years, fluorescence techniques have been improved reaching a nanometer scale resolution (nanoscopy). Fluorescence nanoscopy combined with electron microscopy provides a highly sensitive tool to precisely localize proteins in sub-organelles structures [96]. Immunocytochemical electron microscopy can localize proteins to organelles but technical difficulties are associated with it, including unspecific antibody binding, masked antigen exposure and non-optimal resolution at the sub-organelle level due to the size of the antibodies used. In turn, fluorescence nanoscopy is highly precise to define protein localization at the organelle but not sub-organelle level. A combination of the two methods confers high-resolution and precision. 

### 2.6. Mitochondrial Databases

The availability of the complete genome sequence from bacteria and eukaryotes has been pivotal to define the mitochondrial proteome of mammals. Phylogenetic analyses based on genes present in the mtDNA allowed to ascertain that these genes originated within the α-proteobacteria *Rickettsia*. In addition, these analyses showed that ancestral bacterial genes have been transferred from the mtDNA to the nuclear DNA in some species. Notably, orthologous genes have been detected in the mtDNA in some species and in the nuclear DNA in other species, supporting the common origin from the ancient α-proteobacteria. However, many mitochondrial proteins encoded in the nucleus have no homology with the α-proteobacteria genes, suggesting that these genes originated in a subsequent step of the endosymbiont evolution (reviewed in [97]). 

The creation of databases collecting genomic and proteomic data for mitochondrial proteins has been pivotal for phylogenetic analyses of putative mitochondrial proteins across the various taxonomic ranks. A milestone in defining the mitochondrial proteome of higher eukaryotes is the characterization of the mitochondrial proteome of yeast *Saccharomyces cerevisiae* because its genome is fully sequenced, its cell physiology has been extensively investigated, and because of the evidence that mitochondrial proteins are highly conserved among eukaryotes [97,98]. Indeed, homology comparisons to previously identified yeast mitochondrial proteins remain a powerful tool to define a mitochondrial protein in other eukaryotes. MITOP, the first database containing both nuclear- and mitochondrial-encoded proteins, and their genetic and functional annotation across five mammalian species, was released in 1999 [99]. Pagliarini et al. [100] performed phylogenetic analysis, together with MS and GFP tagging, to identify new proteins belonging to Complex I of the mitochondrial respiratory chain. They were able to link uncharacterized proteins to known mitochondrial pathways by means of shared evolutionary trends and identified a new gene, C8orf38, encoding for a protein of Complex I and mutated in an inherited human disease. 

Bioinformatics approaches can provide an estimate of potential mitochondrial proteins by looking for the presence of mitochondria-targeting motifs. Different algorithms have been developed to predict the presence of the cleavable N-terminal pre-sequence. TargetP-2.0 (http://www.cbs.dtu.dk/services/TargetP; [101]) and MitoFates (http://mitf.cbrc.jp/MitoFates; [102]) are among the most recent algorithms. The first one shows significantly increased accuracy in identifying targeting peptides compared to previous bioinformatics tools [101]. Bioinformatic predictions of mitochondrial targeting sequences, domain conservation analyses, and homology comparisons to previously identified yeast mitochondrial proteins can provide a prediction of mitochondria-localized proteins but, alone, are not sufficient to annotate a protein as mitochondrial.

**Figure 1:**
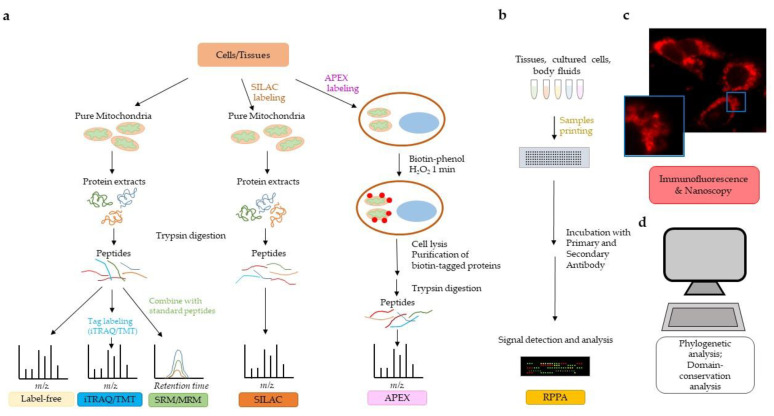
Strategies to investigate the mitochondrial proteome. (**a**) Mass spectrometry (MS) technology allows qualitative and quantitative analysis of the mitochondrial proteome. Different labeling methods can be used to quantify the mitochondrial proteome (see text for details). Label-free and iTRAQ/TMT allow discovery of proteins in a biological sample. SRM/MRM and SILAC are MS technologies that allow proteome quantification. APEX bypasses the need for organelle purification. (**b**) Reverse phase protein array (RPPA) allows for the quantification of proteins from relatively small amounts of biological samples (tissues, cell cultures, body fluids) and requires the availability of highly specific primary antibodies. (**c**) Immunofluorescent microscopy/nanoscopy is widely used to assess and/or confirm mitochondrial localization of proteins. The immunofluorescent image shows human pancreatic cancer cells stained with a primary antibody to pyruvate dehydrogenase followed by Alexa Fluor 568 secondary antibody. Nanoscopy can provide a higher resolution on subcellular compartmentalization than traditional immunofluorescence, as detailed in the text. (**d**) Bioinformatic analyses of mitochondrial targeting sequences, phylogenetic analyses, domain conservation analyses, and homology comparisons to previously identified mitochondrial proteins may predict localization of proteins in mitochondria.

To date, the MitoCarta 2.0 human inventory [103] reports 1158 mitochondrial proteins, predominantly belonging to the matrix, IMM, and IMS. OMM proteins are still under-represented, possibly because many of them may be lost in assays involving mitochondrial purification procedures. The MitoMiner database (mitominer.mrc-mbu.cam.ac.uk) provides a complete, updated compendium of mitochondrial proteins identified in 56 large-scale proteomic datasets from MS and GFP-tagging studies. Overall, fluorescence microscopy of GFP-tagged proteins provides the best evidence of mitochondrial localization and is more reliable than MS alone, because the latter can include false positives due to contamination of the mitochondrial preparation with proteins from other compartments. The advent of more sophisticated methodological approaches, including APEX, is in part solving the issue of false positive in mitochondrial subcompartments. Different strategies used to investigate the mitochondrial proteome are summarized in Figure 1. Methodologies to study the mitochondrial interactome and discussed in this review are not depicted.

## 3. The Mitochondrial Proteome of Tumor Cells

Recent studies have highlighted crucial changes of the mitochondrial proteome in cancer versus normal tissues and in primary cancers versus distant metastases, in a cancer type-dependent manner. A comprehensive analysis of different cancer types demonstrated that poor patients’ survival correlated with inhibition of at least one mitochondrial pathway in 10 out of 15 cancer types [104]. The worst clinical outcome was observed in patients exhibiting downregulation of mitochondrial genes in the primary cancer. This phenotype correlated with the expression of epithelial-to-mesenchymal transition markers, a characteristic of metastatic cells [104]. These results are consistent with previous studies demonstrating that mitochondrial dysfunction is associated with cancer progression to a metastatic phenotype in some cancer types [9,56,57,105]. Here, I will report not a compendium of the studies on the mitochondrial proteome in cancer, but a snapshot on relevant examples of altered mitochondrial proteome in some of the most diagnosed tumor types and discuss potential clinical implications.

### 3.1. Breast Cancer

Breast cancer is the second most common cancer in women (www.cancer.gov). In 2013, a proteomic work analyzed changes in the levels of known mitochondrial membrane proteins during breast cancer progression using parental MCF-10A breast epithelial cells and their derivatives representing three progressive tumor grades of RAS-driven cancer [106]. LC-MS/MS proteomic analysis revealed that all three cell derivatives had a marked reduction of mitochondrial membrane proteins belonging to the mitochondrial respiratory chain. In addition, the levels of VDAC, an OMM protein that functions as gatekeeper for the traffic of metabolites in and out the mitochondria, were also dramatically reduced during malignant transformation. A decrease in the expression of mitochondrial membrane proteins was associated with mitochondrial dysfunction.

Another work found that Adriamycin-resistant MCF-7 breast cancer cells have altered levels of mitochondrial proteins involved in apoptosis, fatty acid oxidation, heme biosynthesis and ATP production compared to Adriamycin-sensitive parental cells [107], suggesting that changes in the abundance of mitochondrial proteins may participate to the mechanism of drug resistance in cancer. Notably, reduction of the levels of mtDNA-encoded proteins caused by depletion or deletions of the mtDNA has also been reported to promote chemoresistance in cancer cells (reviewed in [56]), supporting the notion that mitochondrial changes play a crucial role in modulating cell survival. 

Two-dimensional differential gel electrophoresis and MALDI-TOF MS of mitochondrial proteins purified from the non-tumorigenic breast epithelial cells MCF-10A, the non-invasive breast cancer cell line MCF-7, and the metastatic breast cancer cell line MDA-MB-231 led to the identification of 33 mitochondrial proteins differentially expressed in cancer versus normal cells. Notably, prohibitin, a mitochondrial protein regulating cell proliferation, cristae formation, mitochondrial activity, and the stability of the mitochondrial genome, was present in the mitochondria of normal breast cells but was delocalized to the nuclear compartment in non-invasive and invasive breast cancer cells [108]. Intriguingly, nuclear prohibitin is able to control transcription and cell cycle (reviewed in [109]), suggesting that the delocalization of prohibitin from mitochondria to the nucleus in cancer cells may have broad implications in cellular homeostasis. These studies suggest that nuclear prohibitin may represent a novel diagnostic biomarker in breast cancer and a new potential target of anti-cancer therapies. 

Besides prohibitin that localizes both in the mitochondria and the nucleus, dual localization of other proteins has also been demonstrated. Dual localization of proteins may result from different mechanisms, such as by alternative splicing that produces different isoforms of the protein starting from the same gene, or by use of an alternative starting codon during translation, or by interaction with specific chaperones upon a stimulus/stress. The proto-oncogene c-SRC is an example. The SRC family of non-receptor tyrosine kinases does not contain typical mitochondrial localization signals but localization of these kinases within mitochondria has been documented, indicating that adaptor proteins/chaperons may allow their localization to the mitochondria [74]. Within mitochondria, SRC regulates the activity of several proteins, including enzymes of the mitochondrial respiratory chain and the mitochondrial single-stranded DNA-binding protein involved in mtDNA replication [74,110]. Immunofluorescence analysis has shown that levels and activity of mitochondrial c-SRC are elevated in triple-negative breast cancer cells [110]. Notably, increased levels of intramitochondrial c-SRC kinase result in reduced amount of mtDNA, mitochondrial dysfunction, shorter cell cycle, and increased invasion ability of breast cancer cells [110], suggesting that localization of some proteins into mitochondria may confer invasive ability to cancer cells by affecting the mitochondrial activity and signaling and may represent additional prognostic biomarkers to define cancer aggressiveness.

### 3.2. Prostate Cancer

Prostate cancer is the most common malignancy in men of the Western World. Recent MS advancements have driven the research of potential diagnostic/prognostic prostate cancer biomarkers in body fluids [111,112], primary tumors [113] and the tumor microenvironment [114]. A recent high-throughput MS analysis of clinical tissue specimens from benign prostate hyperplasia (BPH), primary prostate cancer and castration-resistant prostate cancer (CRPC; the most advanced form of prostate cancer) shows that the expression levels of some enzymes of the Krebs’ cycle, including aconitase-2 and oxoglutarate dehydrogenase, are increased in primary cancer versus BPH but decreased during transition from primary, untreated cancer to CRPC. An exception is the enzyme malate dehydrogenase 2, which linearly increased during prostate cancer development and progression [115]. This study indicates that some mitochondrial metabolic enzymes may represent useful clinical biomarkers to trace development and progression of prostate cancer. 

In addition to MS, the RPPA technology has been used to analyze changes in the levels of mitochondrial proteins during prostate cancer progression. Herrmann PC et al. [116] combined laser capture microdissection with RPPA to assess changes in mitochondrial- and nuclear-encoded subunits of cytochrome c oxidase and correlated them with malignant transformation and progression in 30 human prostate tissue specimens. A progressive shift towards nuclear-encoded cytochrome c oxidase subunits IV, Vb, and VIc compared to mitochondrial-encoded cytochrome c oxidase subunits I and II was detected during transition from normal prostate to premalignant lesions and further during progression to invasive cancer [116]. These results suggest that the shift from mitochondria- to nuclear-encoded cytochrome c oxidase subunits may represent a potential prognostic/diagnostic marker for prostate cancer. 

Recently, Altieri’s lab analyzed clinically annotated patient series of primary and metastatic prostate cancer and publicly available genomic databases and found that the MFF gene is amplified in prostate cancer patients, correlating with disease relapse and worst survival [117]. Besides human prostate cancer, the authors observed increased levels of MFF in tissue samples of other tumor types, including non-small cell lung cancer and multiple myeloma. A proteomic MS screening for MFF-interacting proteins in prostate cancer cells revealed a strong interaction of MFF with VDAC1 [118]. Altieri and collaborators analyzed the interface of the MFF-VDAC1 complex and designed a synthetic peptide to counteract the interaction between the two proteins. In preclinical models, treatment with the MFF peptidomimetic displayed anti-cancer activity in patient-derived xenografts, primary breast and non-small cell lung adenocarcinoma organoids, and in glioblastoma neurospheres [118], thus identifying the MFF-VDAC1 complex as an actionable therapeutic target in cancer. 

### 3.3. Ovarian Cancer

In vivo studies have shown that mitochondrial dysfunction is involved in chemoresistance of ovarian cancers [56,119]. Two-dimensional difference gel electrophoresis coupled with MALDI-TOF MS in platinum-sensitive and platinum-resistant ovarian cancer cell lines demonstrated that five enzymes participating in the electron respiratory chain (ATP-α, PRDX3, prohibitin, ETF, ALDH) were downregulated in chemoresistant cells. The downregulation of one of these proteins, prohibitin, was confirmed in vivo in ovarian cancer tissues from patients resistant to platinum-based chemotherapy [120], implicating it as a prognostic novel biomarker of ovarian cancer chemoresistance.

Similarly, SILAC-based proteomic analysis performed in doxorubicin-resistant and doxorubicin-sensitive ovarian cancer cell lines showed that acquisition of chemoresistance is associated with dysregulation of 122 mitochondrial proteins regulating transport, metabolism, energy production, and apoptosis. Among them, several enzymes participating to the electron respiratory chain were significantly downregulated in doxorubicin-resistant cells [121]. Electron microscopy and immunofluorescence confirmed altered mitochondrial morphology and localization in chemoresistant cells [121], again indicating that selected mitochondrial proteins may be used as potential biomarkers of cancer chemoresistance to specific drugs. 

Zhan’s group performed proteomic analysis of mitochondrial preparations from 7 ovarian high-grade cancer tissues and 11 control ovaries with benign gynecological diseases by using iTRAQ and LC-MS/MS [122,123]. Using this approach, they identified 1198 differentially regulated mitochondrial proteins. Among these, nuclear-encoded enzymes participating to glycolysis, Krebs’ cycle and OXPHOS were significantly upregulated in ovarian cancer [123]. Taken together, these studies demonstrate that the mitochondrial proteome is altered in ovarian cancer. Future functional studies are needed to assess the contribution of the altered mitochondrial proteins to the pathology of ovarian cancer and whether these changes may serve as prognostic ovarian cancer biomarkers. 

### 3.4. Colorectal Cancer

A recent study mapped the proteome of metastatic colorectal cancer cells by SILAC labeling following by cell fractionation into cytoplasm, plasma membrane, mitochondria and ER/Golgi, nuclear, chromatin-bound and cytoskeletal proteins, and MS [124]. The analysis revealed an altered expression of proteins of the Krebs’ cycle and OXPHOS. Notably, the authors detected some OXPHOS proteins, including three proteins of the Cytochrome b-c1 mitochondrial complex, in the cytoskeleton compartment of colon cancer cells and a reduction of their expression in metastatic cells. 

A previous proteomic study used MALDI-TOF/TOF MS in total extracts from seven pairs of colorectal cancer tissues and their adjacent normal mucosa and revealed significant changes in the expression of mitochondrial enzymes participating to the Krebs’ cycle, with a 3- to 5-fold increase in the levels of malate dehydrogenase and a 6-fold decrease in the levels of aconitase 2 and aconitate hydratase [125], providing evidence for alteration of metabolic pathways in colorectal tumorigenesis, and highlighting the potential of mitochondrial proteins as diagnostic biomarkers in colorectal cancer. 

### 3.5. Pancreatic Cancer

Pancreatic cancer is one of the cancers with the worst prognosis having a 5-year survival of less than 10%. Isotope-coded affinity tag technology and tandem MS was used in 2005 to perform quantitative proteomic profiling of pancreatic cancer tissues and normal pancreas [126]. Among several proteins deregulated in pancreatic cancer, cytochrome c oxidase and succinate dehydrogenase were significantly downregulated, indicating the reduction of the mitochondrial energy metabolism in pancreatic cancer. Notably, an independent study later found that a combination therapy with CDK4/6 inhibitors may be highly effective in killing pancreatic cancer cells by yielding increased mitochondrial mass and promotion of OXPHOS [127]. This study supports recent evidence that targeting metabolic reprogramming may be an effective combinatorial strategy to kill cancer cells (reviewed in [128]). 

### 3.6. Hematological Tumors

In 2005, a nano-LC-MS/MS experimental approach was used to characterize the mitochondrial proteome of human leukemia cells [129]. In this study, 680 mitochondrial and mitochondria-associated proteins were identified from sucrose gradient-purified mitochondria of Jurkat T leukemia cells. Among these 680 proteins, 227 were known mitochondrial proteins. This work generated the first reference mitochondrial proteome of human leukemia cells.

Immunofluorescence and immunoblotting analysis in myeloid leukemia cells revealed that the stress-activated protein kinase JNK/SAPK can localize into mitochondria following treatment with 12-O-tetradecanoylphorbol-13-acetate, an agent that induces a differentiated monocytic phenotype and apoptosis [130]. Localization of JNK/SAPK in mitochondria upon an apoptotic stress has been confirmed in other cell types (see also [131]).

### 3.7. Other Tumors

Mitochondrial accumulation of epidermal growth factor receptor (EGFR), a receptor tyrosine kinase prevalently localized in the plasma membrane, has been shown to promote metastasis in non-small cell lung carcinomas and to be very high in stage IV [74,132]. A high percentage (40–89%) of non-small cell lung carcinomas shows overexpression of EGFR. It is also overexpressed in many other cancers and its overexpression is associated with poor prognosis. Intriguingly, EGFR contains a mitochondria-localization signal. The first study to show that EGFR localizes also to mitochondria performed confocal microscopy and biochemical fractionation in murine fibroblasts upon EGF stimulation [133]. A subsequent study demonstrated EGFR insertion into the mitochondrial membrane and a role of c-SRC in EGFR translocation to the mitochondria [134]. Within mitochondria, EGFR would affect mitochondrial fission and energy metabolism [133,134], thereby promoting the invasive properties of the cells and representing an adverse prognostic marker in non-small cell lung carcinomas. 

Human neuroblastoma cells display accumulation of activated protein kinase B (AKT) within mitochondria upon activation of PI3-Kinase signaling by insulin or insulin-like growth factor-1 [135]. Active AKT is prevalently cytosolic, but a fraction of active AKT has been also detected in nuclei and mitochondria. Mitochondrial AKT increases in neuroblastoma cells upon stimulation with growth factors but also in the presence of an apoptotic stimulus [135]. In response to staurosporine, neuroblastoma cells expressing mitochondrial-targeted active AKT display decreased levels of apoptotic markers suggesting that localization of AKT within mitochondria may protect neuroblastoma cells from undergoing apoptosis in response to specific stressors [136]. 

Immunofluorescent and immunoblotting analysis of cellular fractions in a variety of neoplastic cells have shown localization of protein kinase C delta (PKCδ), a cytosolic protein, into mitochondria after cell treatment with phorbol esters, oxidants or with anti-cancer drugs such as etoposide and cisplatin. The localization of PKCδ within mitochondria would sustain apoptosis [137,138,139,140], thus it may represent an adverse prognostic marker.

Taken together, these studies, summarized in Table 1, highlight the contribution of qualitative and quantitative changes of the mitochondrial proteome during tumorigenesis, cancer progression and chemoresistance, and define new potential diagnostic and prognostic tumor biomarkers as well as potential target for anti-cancer therapy.

## 4. Conclusions

Mitochondria are very complex organelles that influence tumor biology in several stages, including development, growth, angiogenesis, survival, and progression to an invasive and chemoresistant phenotype [9,56,141]. They can be considered a “signaling hub” that actively communicate with other cell compartments (peroxisomes, nucleus, ER) to shape the fate of the cells [40,56,57,142,143]. 

The dual localization of some proteins in and out the mitochondria is an essential tool to accomplish specific subcellular tasks not only upon stress stimuli, cell transformation, and differentiation, but also during the normal cell homeostasis. For example, the localization of the mitochondrial pyruvate dehydrogenase complex from mitochondria to the nucleus during cell cycle progression generates a nuclear pool of acetyl-CoA used for histone acetylation and S-phase entry [144]. On the other side, delocalization of some proteins in and out mitochondria during cancer development and progression may allow transformed cells to accomplish specific functions, enabling them to strive, proliferate and invade. 

Mitochondrial proteomics, integrated with other omics data, offers the potential to address the multiple functions and roles of mitochondrial biology in normal and cancer cells. Furthermore, a detailed characterization of the changes in the mitochondrial proteome during the different steps of cancer initiation and progression may drive the discovery of novel cancer biomarkers and potential new chemotherapeutic targets. In this context, the gene encoding the anti-apoptotic mitochondrial protein BCL-2 (B-cell lymphoma 2) deserves a special mention. BCL-2 was identified because it is frequently amplified in non-Hodgkin’s B-cell lymphomas via t(14;18) chromosomal translocations that place BCL-2 at 18q21 under the control of potent enhancers of the immunoglobulin heavy-chain locus at 14q32 (reviewed in [145]). The majority of follicular non-Hodgkin’s B-cell lymphomas contain the t(14;18) translocation and highly express BCL-2. Chronic lymphocytic leukemia is also characterized by high levels of BCL-2, though the t(14;18) rearrangment is only occasional in this neoplasm [146]. In 2016, FDA approved the BCL-2 inhibitor Venetoclax for treatment of an aggressive form of chronic lymphocytic leukemia. In 2018-2019, FDA approved Venetoclax for the treatment of small lymphocytic lymphoma and in combination therapy for newly diagnosed acute myeloid leukemia. Clinical trials are currently investigating the use of Venetoclax also for pediatric and adult solid tumors (www.cancer.gov). Venetoclax represents the first mitochondrial/apoptosis-targeting therapeutic agent available to patients. These studies on a mitochondria-localized protein strongly support the translational potential of basic science into clinical applications and the “from bench to bed” philosophy. 

## Figures and Tables

**Table 1:** Summary of mitochondrial proteins/pathways deregulated in tumor cells, as discussed in this review.

Tumor Type	Cells/Tissues Analyzed	Proteins/Pathways Deregulated	References
Breast cancer	MCF-10A MCF-10A-cancer derivatives	Decreased levels of proteins of the respiratory chain	[106]
Breast cancer	Adriamycin-sensitive, and adriamycin-resistant MCF-7 cancer cells	Decreased levels of proteins involved in fatty acid oxidation, apoptosis, heme biosynthesis in Adriamycin-resistant cells	[107]
Breast cancer	MCF-10A MCF-7 MDA-MB-231	Deregulation of mitochondrial proteins involved in several pathways; prohibitin delocalized in the nucleus of cancer cells	[108]
Breast cancer	MCF-10A MCF-12A MCF-7 MDA-MB-231 BT-483 SK-BR-3 MDA-MB-453 BT-549 T47D MDA-MB-468 hTERT-HME-1	High levels of intramitochondrial c-SRC in triple-negative breast cancer cells	[110]
Prostate cancer	Benign prostate hyperplasia (BPH) Primary prostate cancer Castration-resistant prostate cancer (CRPC)	Expression of some enzymes of the Krebs’ cycle, including aconitase-2 and oxoglutarate dehydrogenase, increased in primary cancer versus BPH but decreased in primary cancer versus CRPC Malate dehydrogenase 2 linearly increased during prostate cancer development and progression	[115]
Prostate cancer	Biopsies from normal prostate tissue, premalignant lesions, non-invasive malignant lesions, invasive malignant lesions	A progressive shift towards nuclear-encoded cytochrome c oxidase subunits IV, Vb, and VIc compared to mitochondria-encoded cytochrome c oxidase subunits I and II detected during transition from normal prostate to premalignant lesions and further during progression to invasive cancer	[116]
Prostate cancer	Normal prostate tissues Prostate cancer tissues	Increased levels of the mitochondrial fission factor MFF	[117,118]
Ovarian cancer	Platinum-sensitive and platinum-resistant ovarian cancer cell lines Ovarian cancer tissues from patients sensitive and resistant to platinum-based chemotherapy	Five enzymes participating in the electron respiratory chain (ATP-α, PRDX3, prohibitin, ETF, ALDH) downregulated in chemoresistant cells/tissues	[120]
Ovarian cancer	Doxorubicin-resistant and doxorubicin-sensitive ovarian cancer cell lines	Dysregulation of 122 mitochondrial proteins regulating transport, metabolism, energy production and apoptosis in doxorubicin-resistant cells	[121]
Ovarian cancer	High-grade ovarian cancer tissues Normal ovaries	1198 differentially regulated mitochondrial proteins in cancer. Nuclear-encoded enzymes participating to Krebs’ cycle and OXPHOS significantly upregulated in ovarian cancer	[123]
Colorectal cancer	Metastatic and non- metastatic colorectal cancer cell lines	Altered expression of proteins of the Krebs’ cycle and OXPHOS. Some OXPHOS proteins, including three proteins of the Cytochrome b-c1 mitochondrial complex, detected in the cytoskeleton compartment of colon cancer cells were reduced in metastatic cells	[124]
Colorectal cancer	Colorectal cancer tissues and their adjacent normal mucosa	Changes in the expression of mitochondrial enzymes participating to the Krebs’ cycle, with a 3- to 5-fold increase in the levels of malate dehydrogenase and a 6-fold decrease in the levels of aconitase 2 and aconitate hydratase	[125]
Pancreatic cancer	Pancreatic cancer tissues Normal pancreas	Cytochrome c oxidase and succinate dehydrogenase significantly down-regulated in cancer	[126]
Hematological tumors	Jurkat T leukemia cells	680 mitochondrial and mitochondria-associated proteins were identified This work generated the first reference mitochondrial proteome of human leukemia cells	[129]
Hematological tumors	Myeloid leukemia cells	The stress-activated protein kinase JNK/SAPK localized into mitochondria following treatment with 12-O-tetradecanoylphorbol-13-acetate, an agent that induces a differentiated monocytic phenotype and apoptosis	[130]
Hematological tumors	Multiple myeloma	Upregulation of MFF	[117,118]
Non-small cell lung cancer	Non-small cell lung cancer Normal lung tissue	Upregulation of MFF	[117,118]
Non-small cell lung cancer	Non-small cell lung carcinoma cells and tissues Normal lung tissue	Mitochondrial accumulation of epidermal growth factor receptor (EGFR), a receptor tyrosine kinase prevalently localized in the plasma membrane, promoted metastasis in non-small cell lung carcinomas and was very high in stage IV cancers	[74,132]
Neuroblastoma	Neuroblastoma cells	Accumulation of activated protein kinase B (AKT) within mitochondria upon activation of PI3-Kinase signaling by insulin or insulin-like growth factor-1	[135,136]
Miscellaneous tumor types	U-937 myeloid leukemia cells Breast cancer cell lines Prostate cancer cell lines	Mitochondrial localization of PKCδ after treatment with phorbol esters, oxidants or with anti-cancer drugs, such as etoposide and cisplatin	[137,138,139,140]

## Abbreviations

2-DE: two-dimensional gel electrophoresis; APEX, ascorbate peroxidase; AP-MS, affinity purification and tandem mass spectrometry; DRP1, dynamin-related protein 1; ER, endoplasmic reticulum; ETC, electron transport chain; GFP, green fluorescent protein; IMM, inner mitochondrial membrane; HA, hemagglutinin; IMS, intermembrane space; iTRAQ, isobaric tags for relative and absolute quantitation; LC, liquid chromatography; LC-MS, liquid chromatography coupled with mass spectrometry; MALDI-TOF, Matrix-Assisted Laser Desorption/Ionization-Time Of Flight; MAMs, mitochondria-associated membranes; MDPs, mitochondrial-derived peptides; MFN, mitofusin; MOTS-c, mitochondrial ORF of the 12S ribosomal RNA type-c; MRM, multiple reaction monitoring; MS, mass spectrometry; mtDNA, mitochondrial DNA; m/z: mass-to-charge; OMM, outer mitochondrial membrane; OPA1, optic atrophy 1; ORF, open reading frame; OXPHOS, oxidative phosphorylation; PAGE, polyacrylamide gel electrophoresis; PRM, parallel reaction monitoring; PTM, post-translational modification; ROS, reactive oxygen species; RPPA, Reverse Phase Protein Array; SILAC, stable isotope labeling by amino acids in cell culture; SRM, selected reaction monitoring; TIM, translocase of the inner membrane; TOM, translocase of the outer membrane; TMT, tandem mass tags; UPR, unfolded protein response; XL-MS, cross-linking mass spectrometry.
